# Depletion of Regulatory T Cells in Visceral Adipose Tissues Contributes to Insulin Resistance in Hashimoto's Thyroiditis

**DOI:** 10.3389/fphys.2018.00136

**Published:** 2018-02-28

**Authors:** Min Yang, Li Su, Qin Tao, Chenxi Zhang, Yueyue Wu, Jun Liu

**Affiliations:** ^1^Department of Endocrinology, The Fifth People's Hospital of Shanghai, Fudan University, Shanghai, China; ^2^Testing and Analysis Center, School of Pharmacy, Second Military Medical University, Shanghai, China

**Keywords:** Hashimoto's Thyroiditis, regulatory T cells, insulin resistance, cytokines, visceral adipose tissues

## Abstract

Hashimoto's Thyroiditis (HT) is a common organ-specific autoimmune disorder associated with a high incidence, and insulin resistance is highly related to autoimmune. Here, we examined the insulin sensitivity in HT patients and found decreased insulin sensitivity occurred in HT patients. To explore the relationship between impaired insulin sensitivity and immune status, we established HT model mice which showed similar pathological features and immune features to HT patients. In HT model mice, reinfusion of regulatory T cells (Tregs) from peripheral blood of normal mice could improve insulin sensitivity and decrease the inflammation. Anti-CD25 antibodies blocked beneficial effects from reinfusion of Tregs, but delayed administration of anti-CD25 antibodies could not abolished the effect from Tregs. Delayed administration of anti-CD25 antibodies abolished exogenous Tregs in peripheral blood, but there were increased exogenous Tregs located to visceral adipose tissues (VATs) which modulated the expression of cytokines in VATs. These findings suggest that insulin resistance exists in HT patients and it associates with the decreased Tregs and increased inflammation in the VATs.

## Introduction

Hashimoto's Thyroiditis (HT), a common organ-specific autoimmune disorder, presents the infiltration of the thyroid gland by inflammatory cells and the production of autoantibodies to thyroid-specific antigens (Ajjan and Weetman, 2015). HT is associated with the destruction of thyroid cells, and hypothyroidism is the main clinical manifestation (Işgüven et al., 2016). Hypothyroidism is associated with the susceptibility of insulin resistance and metabolic syndromes (Fernandez-Real et al., 2006; Roos et al., 2007; Jornayvaz et al., 2012). So HT should be connected to insulin resistance directly. But the reason that induces insulin resistance in HT patients is not clear.

Regulatory T cells (Tregs) are an important class of T cells maintaining immune self-tolerance (Yu et al., 2017). Tregs help to prevent the immune response against self-antigens (Perdigoto et al., 2015). In HT, T cells destruct the thyroid epithelial cells and thyroid epithelial structure (Stassi and De Maria, 2002). The role of Tregs in HT has been reported by many researchers that the thyroid damage in HT relating to Tregs/Th17 cell imbalance (Kristensen, 2016). Foxp3^+^ Tregs have the characteristic phenotype (CD4^+^CD25^bright^) and the suppressive effect through the synthesis of TGF, IL-10, and IL-35 (Rodriguez-Munoz et al., 2016). Decreased level of CD4^+^CD25^bright^Foxp3^+^ Tregs associates with increased autoimmune response in HT patients, and the decease of Tregs is also determined in the peripheral blood mononuclear cells (PBMCs) in HT patients in comparison to healthy individuals (Rodriguez-Munoz et al., 2016). It means that the level of Tregs is changed not only in thyroid. Abnormal level of Tregs is another important factor relating to insulin resistance. The percentage of peripheral CD4^+^CD25^+^Foxp3^+^ Tregs is decreased in the patients with type 2 diabetes mellitus and the accumulation of Tregs in adipose tissue plays an important role in reducing obesity related insulin resistance in mice (Yuan et al., 2018). Thus, further researches are required to investigate the relationship between abnormal level of Tregs in HT and insulin resistance.

Experimental autoimmune thyroiditis (EAT) can be induced by the injection of thyroglobulin and adjuvant, and high iodide ingestion can also accelerate the incidence and severity of EAT (Lira et al., 2005; Fang et al., 2007). CBA/J mice are susceptible to EAT (Fang et al. 2007), and the thyroid lesions in this model are similar to the HT.

In this study, we elucidated the role of CD4^+^CD25^+^Foxp3^+^ Tregs to insulin resistance in a CBA/J mice model of HT is established by the administration of iodine and induction of immune response to thyroid globulin. We also investigated the mechanism by which CD4^+^CD25^+^Foxp3^+^ Tregs decreases insulin resistance.

## Materials and methods

### Animals

Female CBA/J mice (6 week aged) were purchased from Shanghai SLAC Laboratory Animal Co., Ltd. (Shanghai, China). Mice were maintained in a specific pathogen-free facility and were cared for in accordance with animal guidelines. The study was approved by the Institutional Animal Care and Use Committee in Second Military Medical University.

### Human serum

For human serum samples, written informed consent was obtained from all participants, and the study was approved and supervised by the Ethics Committee of our hospital. HT patients were first diagnosed and they had not received treatment when we collected the serum. Other basic information of patients is listed in schedule 1.

### HT model of mice

Twenty-three female CBA/J mice of were randomly divided into the control group (*n* = 8) and the high iodine feeding group (*n* = 45) after 1 week of adaptive feeding. The high iodine group was fed with iodine containing 0.05% sodium iodide (1000HI, equivalent to 1,000 times that of the iodine intake of the normal mice), the control group was fed with sterile distilled water. Iodine is immediately used to avoid light preservation. The first immunization and repeated immunization: high iodine group was treated porcine thyroglobulin (mTg) (200 μg/mouse) subcutaneous injection in d0 and d14. The first immunization was used complete Freund's adjuvant (CFA) and repeated immunization was used incomplete Freund adjuvant (IFA).

Sodium iodide was purchased from Shanghai Xinping Fine Chemicals Co. Ltd. Incomplete Freund adjuvant and complete Freund's adjuvant was purchased from Shanghai Tongyi Biotechnology Company. Porcine thyroglobulin (mTg) was purchased from Nanjing Jiancheng Biological Engineering Institute.

### Cytokines assay

TNF-α, IFN-γ, IL1, IL6, IL10, and IL17 was analyzed by a LEGEND plexTM kit (Biolegend, San Diego, CA) according to the manufacturer's protocol.

### Separation of visceral adipose tissue cells

Visceral adipose tissue (VAT) separated and digested with 2 mg/mL collagenase type V in Hanks' balanced salt solution for 30 min at 37°C. Digests were passed through a 40-μm cell strainer.

### Flow cytometry

T cells were stained with fluorochrome-conjugated monoclonal antibodies: anti-mouse CD3, CD4, CD8, IFN-γ, IL-4, IL-17a, CD25, Foxp3 (eBioscience, USA).

PBMC were separated from blood of participants, and then treated with PMA (50 ng/ml), ionomycin (1 μg/ml), BFA (3 μg/ml), and monensin (1.4 μg/ml) for 5 h. Then, those samples were stained with antibodies to different markers for flow cytometry.

Cells were analyzed with the FACS Calibur flow cytometer (BD Biosciences, USA).

### Glucose tolerance assay and insulin assay

At 16 weeks, mice were fasted for 8 h and received intraperitoneal injection of glucose. Serum levels of glucose and insulin in mice were measured at 30, 60, 90, 120 min after the intraperitoneal injection of 20% glucose solution (0.2 ml/10 g weight). Glucose assay kits and mice insulin ELISA kits were purchased from Shanghai Tongyi Biotechnology Company and the analysis was accord to the manufacturer's protocols. Glucose was purchased from Sinopharm Chemical Reagent Company.

### Histological analysis

Sections (5 μm) of formalin-fixed tissue sections were stained with hematoxylin and eosin according to standard procedures. Sections were incubated in 0.3% H_2_O_2_, and followed by another 30 min in 1% BSA. Then, sections were incubated with anti-myeloperoxidase (MPO) (Biocare Medical, USA) primary antibodies overnight at 4°C. Vectastain Elite ABC Staining Kit and DAB Peroxidase Substrate Kit (Vector Laboratories, USA) were used to visualize the staining according to the manufacturer's instructions.

### Thyroid globulin antibodies and anti-thyroid microsomal antibodies assay

The ELISA kits of thyroid globulin antibody (TGAb) and anti-thyroid microsomal antibody (TMAb) were purchased from the Institute of bioengineering in Nanjing.

### Treg cells preparation and injection

At first, B cells, macrophages, CD8^+^ T cells, NK cells, dendritic cells, erythrocytes, and granulocytes were removed from plasma of normal mice by mouse CD4^+^ negative selection kit (Dynal Biotech). Purified CD4^+^ T cells were incubated with phycoerythrin-labeled anti-CD25 anti-body and anti-phycoerythrin magnetic beads and isolated using a MACS separation column (Miltenyi Biotec) to obtain CD4^+^CD25^+^ T cells. Cell purity was assessed by fluorescence-activated cell sorter and was consistently higher than 90% with an average purity of 96% for caudal vein injection (10^6^ cells per mouse). Flow cytometry analysis also confirmed that about 85% purified cells were Foxp3^+^.To mark transferred cells, we mixed lentivirus expressing GFP with purified Tregs (MOI = 6) for 4 h at 37°C in cell culture incubator, and washed with PBS before the injection.

### Treg cells depletion

To deplete Treg cells, mice were given anti-CD25 (Bio X cell (West Lebanon, NH), 50 μg/kg) intravenously 1 h after the reinfusion of Tregs.

### Statistical analysis

The data are shown as mean ± SEM. Data from two groups were compared with an unpaired *t*-test. Data from three or more groups were analyzed with one-way ANOVA with Tukey's multiple comparisons test. *p*-values < 0.05 and *p*-values < 0.01 were considered as statistically significant.

## Results

### Abnormal insulin sensitivity and peripheral immune status are associated with HT patients

**Figure 1:**
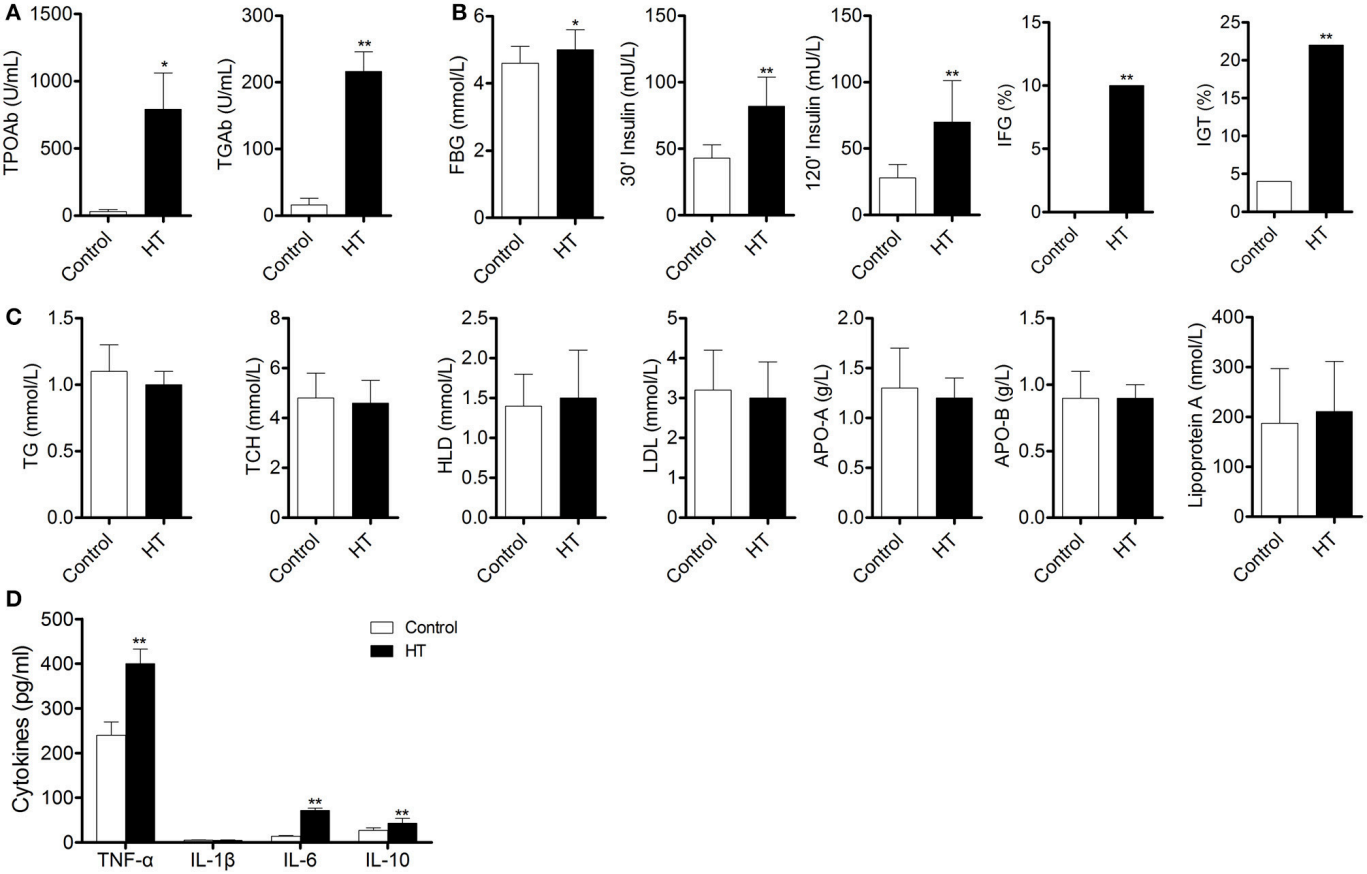
Abnormal insulin sensitivity and peripheral immune status are associated with HT patients. **(A)** Autoantibodies to thyroid peroxidase and thyroid globulin increased in HT patients (^*^p < 0.05; ^**^p < 0.01 compared with normal control). **(B)** Insulin sensitivity decreased in HT patients compared with normal control (^*^p < 0.05; ^**^p < 0.01 compared with normal control). **(C)** Lipid metabolism was not changed between HT patients and normal control. **(D)** Expression pattern of cytokines was changed between HT patients and normal control (^**^p < 0.01 compared with normal control).

To confirm our hypothesis, we analyzed indexes of insulin sensitivity in HT patients (Table 1). These patients had normal thyroid function, but they had enlarged thyroid and increased autoantibodies to thyroid peroxidase and thyroid globulin (Figure 1A, *p* < 0.05). In these patients, fasting blood glucose was raised significantly compared with normal control, and they also had higher postprandial plasma insulin after 30 min or 120 min (Figure 1B, *p* < 0.05). Rates of impaired fasting glucose and impaired glucose tolerance were also significantly higher than normal control (Figure 1B). All the results implied that insulin resistance occurred in HT patients. Serum total triglyceride, total cholesterol, high density lipoprotein (HDL), low density lipoprotein (LDL), apolipoprotein A, apolipoprotein B, and lipoprotein A showed not difference between HT patients and normal control (Figure 1C, *p* > 0.05), which meant the insulin resistance should not relate to fat or lipid metabolism in HT patients.

**Table 1:** Basic information of participants.

	**HT group**	**Control**	***P***
*n*	61	38	
Sex (Female/male)	59/2	37/1	NS
Age (year)	47.2 ± 14.7	40.6 ± 16.9	NS
Hypertension (%)	1.6	0	NS
FT_3_ (pg/ml)	2.9 ± 0.6	2.9 ± 0.7	NS
FT_4_ (ng/dl)	1.1 ± 0.3	1.1 ± 0.1	NS
TT_3_ (ng/ml)	1.1 ± 0.4	1.4 ± 1.5	NS
TT_4_ (ug/dL)	8.5 ± 3.0	9.3 ± 1.7	NS
TSH (uIU/ml)	2.1 ± 1.2	2.2 ± 1.3	NS
TPOAb (U/ml)	790.1 ± 572.3	30.3 ± 16.6	<0.0001
TGAb (U/ml)	216.5 ± 29.3	16.1 ± 9.7	<0.0001

**Figure 2:**
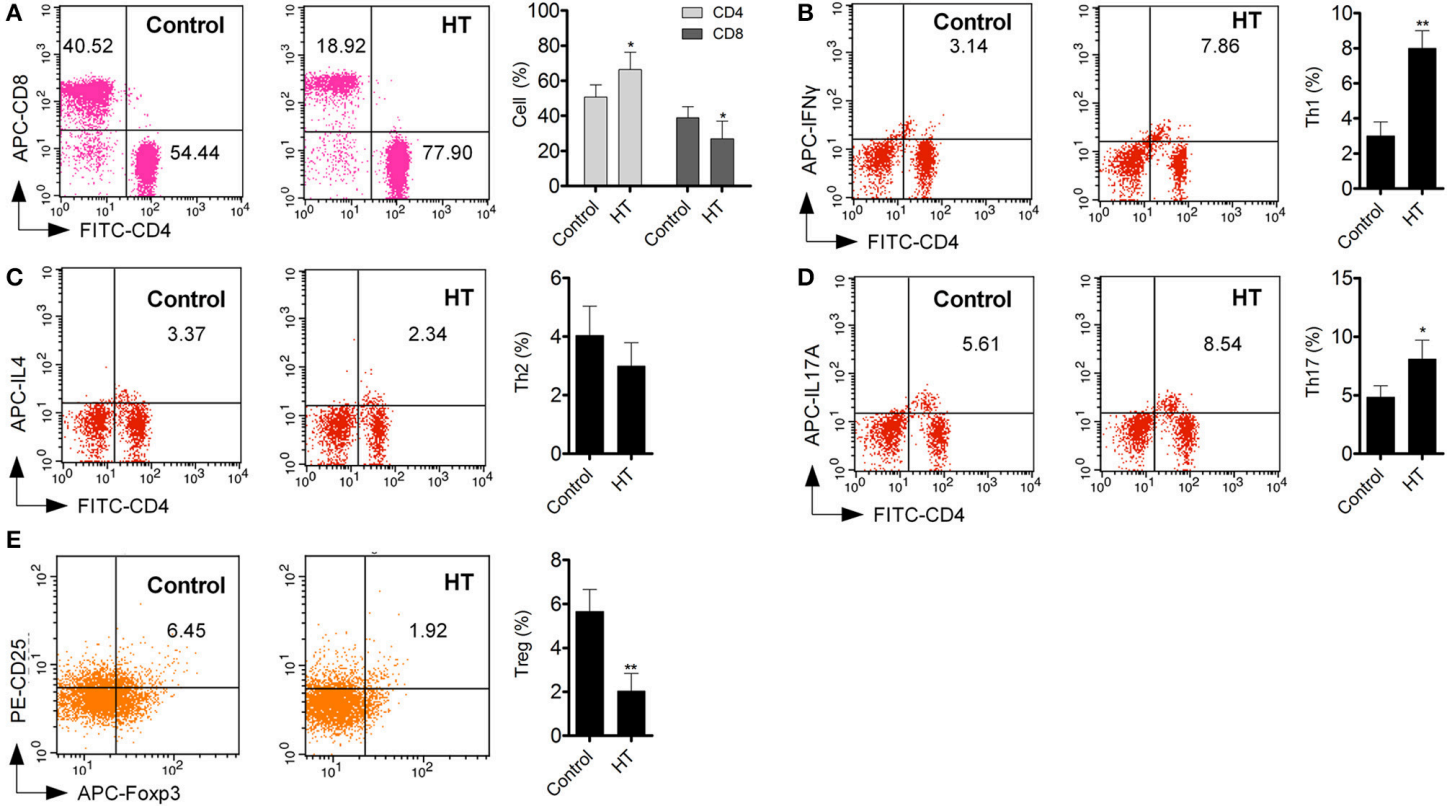
T cell subsets in peripheral blood were different between HT patients and normal control. **(A)** Increased CD4^+^ T cell and deceased CD8^+^ T cells were found in peripheral blood from HT patients (^*^p < 0.05 compared with normal control). **(B–E)** Increased Th1 and Th17 cells were found in HT patients, but Tregs were decreased in HT patients (^*^p < 0.05; ^**^p < 0.01 compared with normal control).

Production of cytokines and subsets of T cell subsets were also measured in peripheral blood from HT patients. Inflammatory cytokines, such as TNF-α, IL-1β, and IL-6 (Figure 1D, *p* < 0.05), were increased in HT patients. IL-10, an important anti-inflammatory cytokine was also changed significantly (Figure 1D, *p* < 0.05). Increased CD4^+^ T cells and decreased CD8^+^T cells were found compared with normal control (Figure 2A). Although Th2 cells did not significantly changed (Figure 2C), Th1 cells and Th17 cells increased in HT patients (Figures 2B,D, *p* < 0.05). CD25^+^Foxp3^+^ Tregs were also decreased in HT patients (Figure 2E, *p* < 0.05).

So insulin resistance and Tregs decrease occurred in HT patients.

### Peripheral CD25^+^Foxp3^+^ tregs from normal mice decreased symptoms of ht in HT model mice

**Figure 3:**
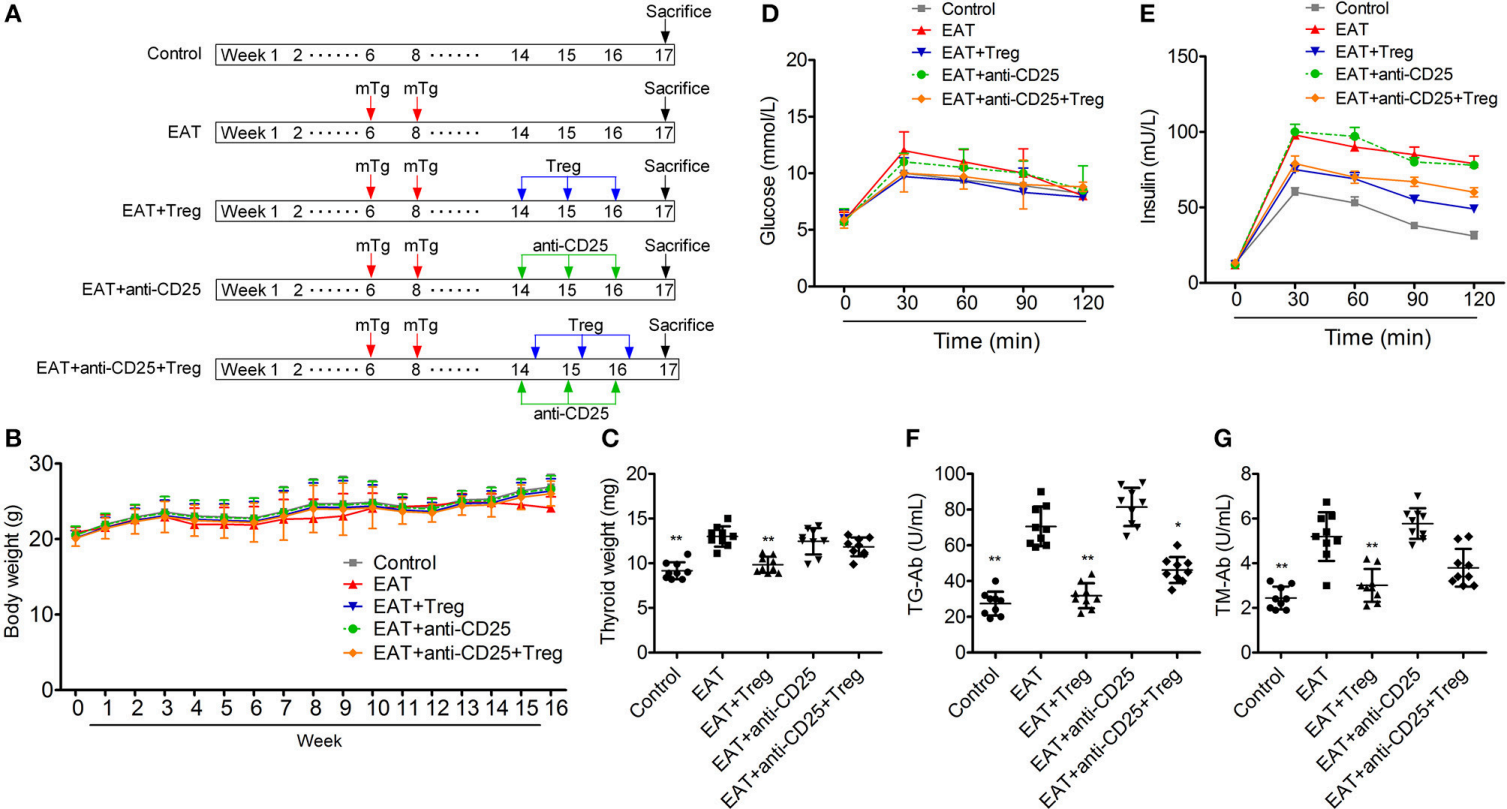
Establishment of HT model mice and the insulin sensitivity of HT model mice was changed. **(A)** The progress of experiment design. **(B)** Bodies weight did not change between groups. **(C,F,G)** Thyroid weight was increased in HT model mice, and the reinfusion of Tregs improved the pathological changes (^*^p < 0.05; ^**^p < 0.01 compared with EAT group). **(D,E)** Insulin sensitivity of HT model mice was impaired compared with control mice.

Figure 3A is the progress of experiment design.

**Figure 4:**
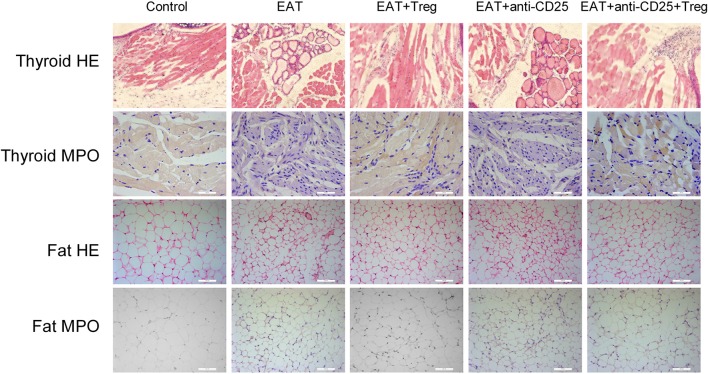
Pathological changes were observed in HT model mice and the reinfusion of Tregs showed beneficial effects to HT model mice.

In HT model mice, autoantibodies to thyroid globulin and thyromicrosome were significantly induced (Figures 3F,G, *p* < 0.05). Infiltration of inflammatory cells to the thyroid gland was also observed in HT model mice in comparison with control mice (Figure 4). So the HT model mice established by us shown the similar pathological features in clinic. Beside the change in the thyroid gland, pathological changes in VATs were also decreased (Figure 4).

The reinfusion of peripheral CD25^+^Foxp3^+^ Tregs from normal mice significantly decreased autoantibodies to thyroid globulin and thyromicrosome and thyroid weight (Figures 3C,F,G). Exogenous CD25^+^Foxp3^+^ Tregs also decreased the infiltration of inflammatory cells to the thyroid gland (Figure 4). These effects could be blocked by the administration of anti-CD25 antibodies (Figures 3C,F,G, 4). It shown the fact that recovery of Tregs in HT model mice modulated the immune system and decreased severity of decrease.

### Changed β-cell function and insulin sensitivity were detected in HT model mice

Although similar glucose concentrations were found between HT model mice and control mice (Figure 3D), higher insulin levels at 30 and 120 min during oral glucose tolerance test were presented in HT model mice comparing with control mice (Figure 3E, *p* < 0.05). High levels of InsAUC30/GluAUC30 and InsAUC120/GluAUC120 were detected in HT model mice (Figures 3D,E, *p* < 0.05), which meant the early phase insulin release and total insulin release were both significantly changed in comparison with control mice. In each group, body weight was not significantly changed (Figure 3B), and it meant the change of insulin sensitivity should not be the result of weight/fat. Thyroid weight increased significantly in HT model mice (Figure 3C), and it might exclude the influence of hypothyroidism which was reported to relate to insulin resistance. These results suggested that the sensitivity of insulin resistance was lacked in HT model mice.

### T cell subsets in peripheral blood and visceral adipose tissues were changed in HT model mice

**Figure 5:**
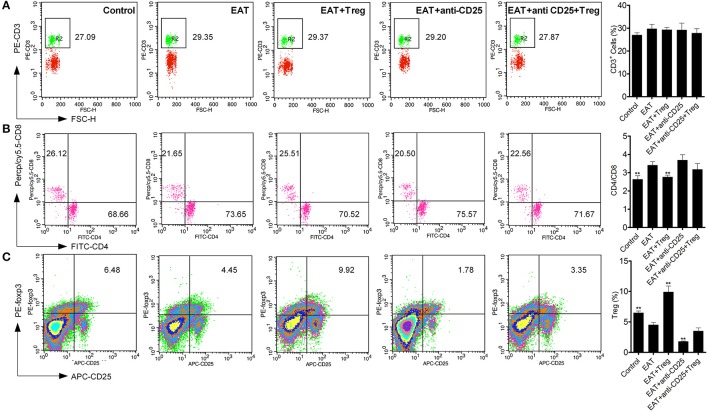
T cell subsets were changed in peripheral blood from HT model mice. **(A)** CD3^+^ cells were not changed in each groups. **(B)** Higher ratio of CD4^+^/CD8^+^ was found in HT model mice and the reinfusion of Tregs rescued imbalance of CD4^+^/CD8^+^ (^**^p < 0.01 compared with EAT group). This beneficial effect was blocked by anti-CD25 antibodies. **(C)** CD25^+^Foxp3^+^ Tregs decreased in peripheral blood from HT model mice and they were rescued by the reinfusion of Tregs, but it was abolished by anti-CD25 antibodies (^**^p < 0.01 compared with EAT group).

As an autoimmune disease, abnormal distribution of T cell subsets is important to HT disease. We analyzed T cells in peripheral blood by flow cytometry, and we found the CD3^+^ cells were not changed between HT model mice and control mice (Figure 5A). The ratio of CD4^+^/CD8^+^ T cells was significantly increased in HT model mice (Figure 5B), which reflects the abnormal immune reaction in HT model mice, and decreased CD25^+^Foxp3^+^ Tregs was also found in peripheral blood of HT model mice (Figure 5C). And these changes were similar to the results of HT patients.

**Figure 6:**
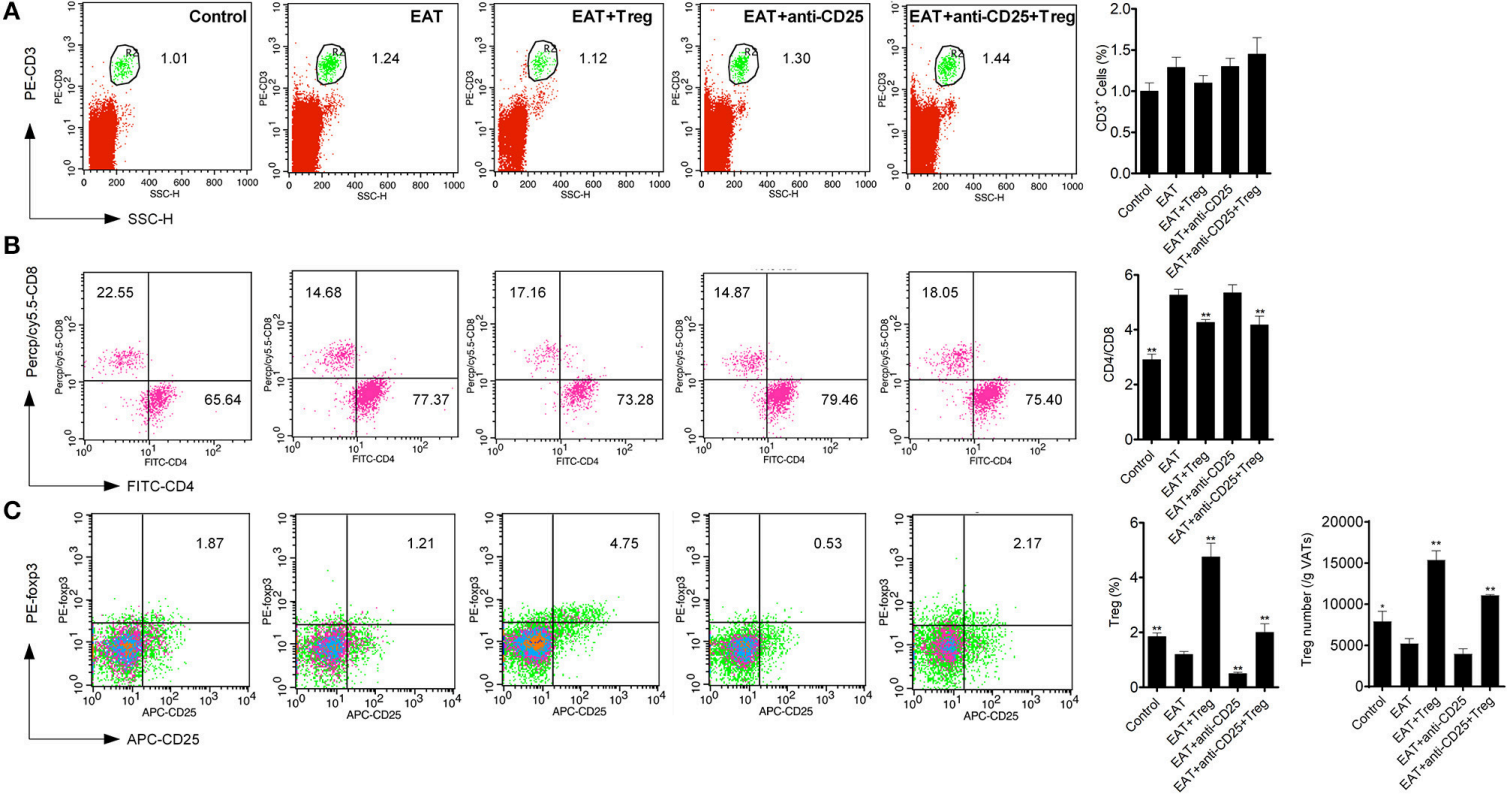
T cell subsets were changed in visceral adipose tissues from HT model mice. **(A)** CD3^+^ cells were not changed in each groups. **(B)** Higher ratio of CD4^+^/CD8^+^ was found in HT model mice and the reinfusion of Tregs rescued imbalance of CD4^+^/CD8^+^ (^**^p < 0.01 compared with EAT group). This beneficial effect was blocked by anti-CD25 antibodies. **(C)** CD25^+^Foxp3^+^ Tregs decreased in visceral adipose tissues from HT model mice and they were rescued by the reinfusion of Tregs, and it was partly abolished by anti-CD25 antibodies (^**^p < 0.01 compared with EAT group; ^*^p < 0.01 compared with EAT group).

Visceral adipose tissues (VATs) are important to insulin sensitivity in physiological condition. We separated the VATs from both of HT model mice and control mice, and the abnormal distribution of T cell subsets was also found in adipose tissues from HT model mice (Figure 6). Increased ratio of CD4^+^/CD8^+^ T cells (Figure 6B) and decreased CD25^+^Foxp3^+^ Tregs (Figure 6C) meant that HT might induce abnormal immune reaction not only in thyroid gland, periphery but also VATs. VATs Tregs are necessary for restore the insulin sensitivity in type-2 diabetes, so decreased VATs Tregs in HT model might associate with the decrease of insulin sensitivity in HT model mice.

### Peripheral CD25^+^Foxp3^+^ tregs from normal mice restored the insulin sensitivity in HT model mice

In HT model mice, CD25^+^Foxp3^+^ Tregs were decreased significantly in both of peripheral blood and VATs when compared with the control group (Figure 6C), and Tregs in VATs are important to insulin sensitivity. Therefore, we separated and transfused peripheral blood CD25^+^Foxp3^+^ Tregs from normal mice into HT model mice, and the treatment significantly increased CD25^+^Foxp3^+^ Tregs in both of peripheral blood and VATs (Figures 5C, 6C). In HT model mice, the transfusion of CD25^+^Foxp3^+^ Tregs decreased the level of InsAUC30/GluAUC30 and InsAUC120/GluAUC120 (Figures 3D,E). All of these results showed that increased level of CD25^+^Foxp3^+^ Tregs not only improved the state of HT but also the sensitivity of insulin. These beneficial effects were abolished by anti-CD25 antibodies (Figure 3E). So CD25^+^Foxp3^+^Tregs related to the insulin resistance in HT model mice.

### Increased CD25^+^Foxp3^+^ tregs in visceral adipose tissues restored the insulin sensitivity in HT model mice

**Figure 7:**
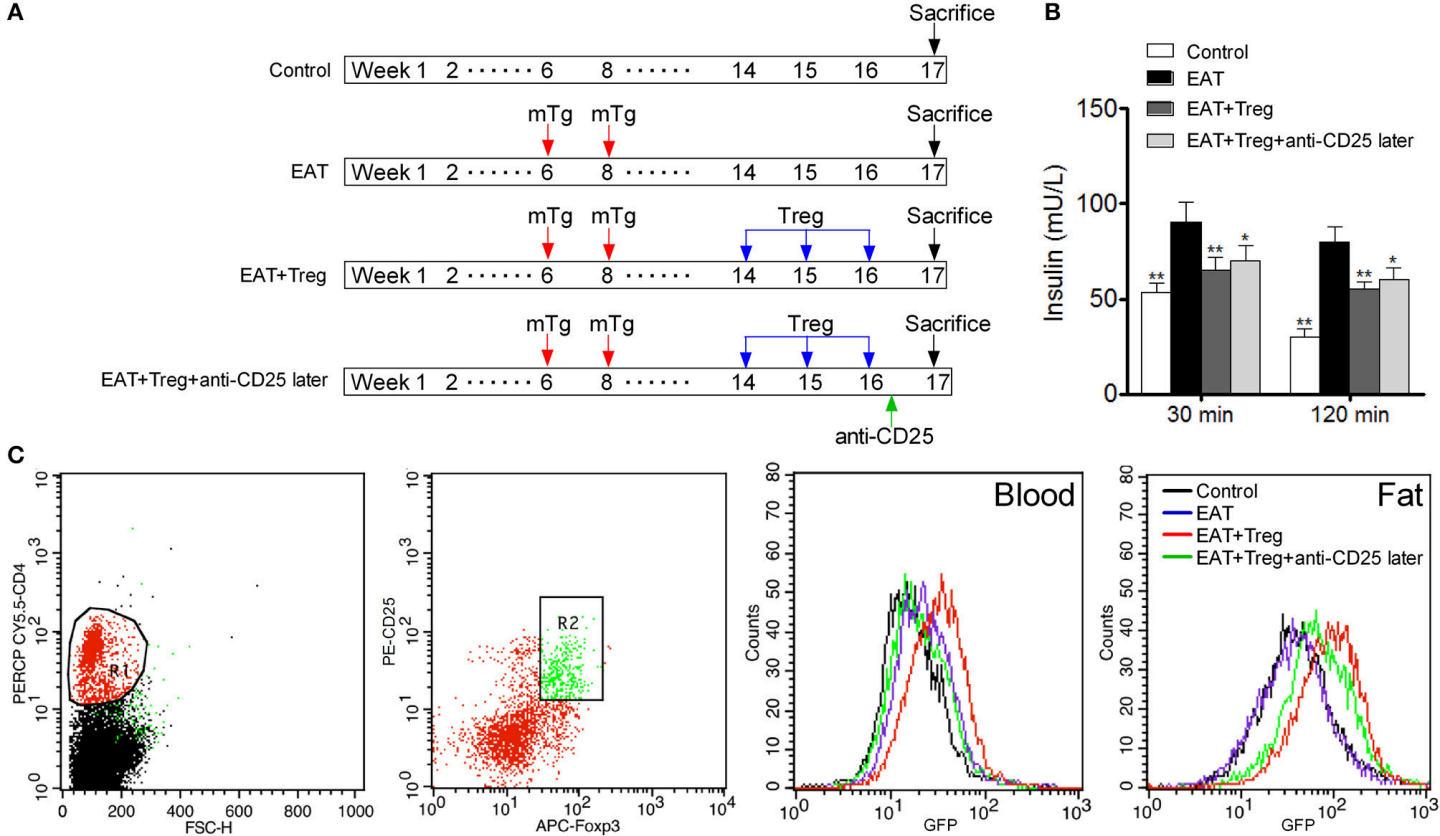
Administration of anti-CD25 antibodies after the reinfusion did not abolished the beneficial effects from exogenous Tregs. **(A)** Design of the experiment. **(B)** Insulin sensitivity was rescued even combined with anti-CD25 antibodies (^*^p < 0.05; ^**^p < 0.01 compared with EAT group). **(C)** We analyzed T cell subsets which were CD4^+^CD25^+^Foxp3^+^ Tregs, and delayed administration of anti-CD25 antibodies decreased exogenous Tregs in peripheral blood but not in visceral adipose tissues.

To confirm the mechanism of CD25^+^Foxp3^+^ Tregs increased insulin sensitivity, we adopted CD25^+^Foxp3^+^ Tregs to HT model mice and administered mice with anti-CD25 antibodies after 3 days (Figure 7A). Administration of anti-CD25 antibodies just partly abolished beneficial effects from CD25^+^Foxp3^+^ Tregs (Figure 7B), so we hypothesized that CD25^+^Foxp3^+^ Tregs in VATs might more important to insulin resistance in HT. To confirm the hypothesis, we expressed GFP in separated CD25^+^Foxp3^+^ Tregs (named GFP-CD25^+^Foxp3^+^ Tregs), and we transfused these GFP-CD25^+^Foxp3^+^ Tregs into HT model mice. After 3 days, mice were treated with anti-CD25 antibodies, and we found almost no GFP-CD25^+^Foxp3^+^ Tregs were detected in peripheral blood after the administration of anti-CD25 antibodies (Figure 7C). But amount of GFP-CD25^+^Foxp3^+^ Tregs could still be detected in VATs (Figure 7C). These adoptive transferred Tregs trafficked to adipose tissue and might played roles in local tissues.

### Cytokines in visceral adipose tissues played important roles in insulin sensitivity in HT model mice

**Figure 8:**
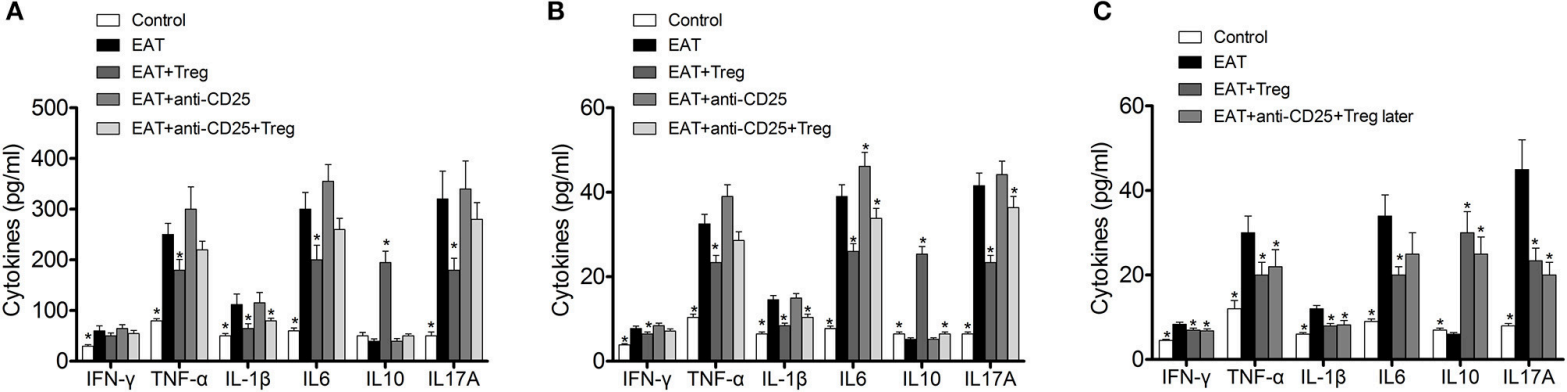
Reinfusion of Tregs changed the pattern of cytokines in both of peripheral blood and visceral adipose tissues. **(A)** Exogenous Tregs normalized the pattern of cytokines in peripheral blood of HT model mice, but it could be blocked by anti-CD25 antibodies (^*^p < 0.05 compared with EAT group). **(B)** Exogenous Tregs normalized the pattern of cytokines in visceral adipose tissues of HT model mice, but it could be blocked by anti-CD25 antibodies (^*^p < 0.05 compared with EAT group). **(C)** Exogenous Tregs normalized the pattern of cytokines in visceral adipose tissues of HT model mice, and it could not be blocked by delayed administration of anti-CD25 antibodies (^*^p < 0.05 compared with EAT group).

In previous researches, cytokines and inflammation in adipose tissue are important to insulin resistance in T2DM, so changed level of CD25^+^Foxp3^+^ Tregs might relate to insulin resistance through cytokines in HT. we measured the cytokines in peripheral blood and VATs, and significantly increased IFN-γ, IL-1β, IL17A, IL6, and TNF-α were found in HT model mice (Figure 8A, *p* < 0.05 compared with control mice). After the transfusion of CD25^+^Foxp3^+^ Tregs, IFN-γ, IL-1β, IL17A, IL6, and TNF-α were decreased and IL-10 was increased significantly (Figure 8A, *p* < 0.05). Combined with anti-CD25 antibodies, the transfusion of CD25^+^Foxp3^+^ Tregs could not induce beneficial effects on the production of cytokines in peripheral blood (Figure 8A).Anti-CD25 antibodies abolished changes of cytokines in VATs when combined anti-CD25 antibodies with CD25^+^Foxp3^+^ Tregs (Figure 8B), but changes of cytokines in VATs still existed when anti-CD25 antibodies were administrated 3 days after the reinfusion (Figure 8C). The production of cytokines was consistent with insulin sensitivity in mice. So Tregs from donor mice should move to VATs and suppressed the local inflammation, which leaded to the reversal of insulin resistance.

## Discussion

HT is an autoimmune disease, and there are growing evidences that autoimmune links to insulin resistance. Although hypothyroidism is thought the major reason that links HT to insulin resistance (Fernandez-Real et al., 2006), the abnormal immune in HT might relate to insulin resistance directly. In this study, we provided that decreased level of CD25^+^Foxp3^+^ Tregs in HT model mice related to increased early phase and total insulin secretion, and the reinfusion of CD25^+^Foxp3^+^ Tregs separated from peripheral blood of normal mice restored the insulin sensitivity in HT model mice. It indicated that decreased Tregs in HT patients might be a critical factor for insulin resistance. We also depleted the peripheral CD25^+^Foxp3^+^ Tregs after the reinfusion, but the effect of insulin sensitivity was still retained. We found the administration of anti-CD25 antibodies depleted CD25^+^Foxp3^+^ Tregs effectively in peripheral blood but not in VATs, and the change of cytokines in peripheral blood and VATs were similar to the results of CD25^+^Foxp3^+^ Tregs. Thus, HT is connected with insulin resistance which relates to the depletion of Tregs in VATs.

Insulin resistance is the excessive insulin accumulation in blood with normal blood glucose levels (Reilly and Saltiel, 2017). Various diseases are associated with insulin resistance, such as type 2 diabetes, metabolic syndromes, obesity, and so on. Most of these diseases are related to abnormal immune responses, and chronic inflammation is an important factor of insulin resistance (Samuel and Shulman, 2012). In type 2 diabetes, it is clear that inflammation in adipose tissue is important to insulin resistance and the pathogenesis, and induction of Tregs alleviates insulin resistance in T2DM mice(Ilan et al., 2010; Nekoua et al., 2016). In HT, auto-reactive CD4^+^ T cells are activated and induced cytotoxic T cells to destruct thyroid cells, and HT is regarded as primarily a T-cell mediated disease (Kristensen, 2016). Tregs are reduced in HT and the existence of Tregs is important to induce tolerance to autoimmune thyroiditis (Xue et al., 2015; Li et al., 2016). But the relationship between abnormal Tregs and insulin resistance in HT has not been reported in HT. The findings of the current study are similar with previous researches. Tregs were decreased significantly in peripheral blood of HT model mice, and the decrease of Tregs in VATs revealed the involvement of Tregs in HT model mice with insulin resistance. Fat-reside Tregs and the chronic adipose inflammatory are related to insulin resistance in patients with type 2 diabetes, and Tregs are important negative regulators of VAT inflammation and insulin resistance (Bapat et al., 2015; Sepehri et al., 2017). Increased level of inflammatory cytokines, such as IFN-γ, IL-1β, IL17A, IL6, and TNF-α, is also related to insulin resistance, and increased anti-inflammatory cytokines can improve glucose metabolism which regulates insulin sensitivity (Winer et al., 2009; Chng et al., 2015). Th1 and Th2 CD4 cells, as well as IFN-γ+CD8 T cells, present in the adipose tissue and the balance of these subsets is associated with not only the responses to antigenic stimulation but also the insulin resistance (McLaughlin et al., 2014; Stafeev et al., 2017).

In conclusion, insulin resistance exists in both of HT patients and HT model mice, and the abnormal distribution of T cell subsets especially in VATs contributes to the insulin resistance in HT.

## Author contributions

All authors have seen and approved the final version of the manuscript. JL conceived and designed the experiments. MY and LS contributed equally to the paper. MY performed the animal experiments and staining of tissues. LS and CZ separated and analyzed T cells from peripheral blood and visceral adipose tissues. YW and QT analyzed cytokines and data. JL wrote the paper. MY and LS also contributed to the writing of the paper.

### Conflict of interest statement

The authors declare that the research was conducted in the absence of any commercial or financial relationships that could be construed as a potential conflict of interest.
